# Guanidinium Toxins and Their Interactions with Voltage-Gated Sodium Ion Channels

**DOI:** 10.3390/md15100303

**Published:** 2017-10-13

**Authors:** Lorena M. Durán-Riveroll, Allan D. Cembella

**Affiliations:** 1 CONACYT—Instituto de Ciencias del Mary Limnología, Universidad Nacional Autónoma de México, Mexico 04510, Mexico; 2 Alfred-Wegener-Institut, Helmholtz Zentrum für Polar-und Meeresforschung, 27570 Bremerhaven, Germany; Allan.Cembella@awi.de

**Keywords:** saxitoxin (STX), paralytic shellfish toxin (PST), tetrodotoxin (TTX), guanidinium, neurotoxin, voltage-gated sodium channels, ion channels

## Abstract

Guanidinium toxins, such as saxitoxin (STX), tetrodotoxin (TTX) and their analogs, are naturally occurring alkaloids with divergent evolutionary origins and biogeographical distribution, but which share the common chemical feature of guanidinium moieties. These guanidinium groups confer high biological activity with high affinity and ion flux blockage capacity for voltage-gated sodium channels (Na_V_). Members of the STX group, known collectively as paralytic shellfish toxins (PSTs), are produced among three genera of marine dinoflagellates and about a dozen genera of primarily freshwater or brackish water cyanobacteria. In contrast, toxins of the TTX group occur mainly in macrozoa, particularly among puffer fish, several species of marine invertebrates and a few terrestrial amphibians. In the case of TTX and analogs, most evidence suggests that symbiotic bacteria are the origin of the toxins, although endogenous biosynthesis independent from bacteria has not been excluded. The evolutionary origin of the biosynthetic genes for STX and analogs in dinoflagellates and cyanobacteria remains elusive. These highly potent molecules have been the subject of intensive research since the latter half of the past century; first to study the mode of action of their toxigenicity, and later as tools to characterize the role and structure of Na_V_ channels, and finally as therapeutics. Their pharmacological activities have provided encouragement for their use as therapeutants for ion channel-related pathologies, such as pain control. The functional role in aquatic and terrestrial ecosystems for both groups of toxins is unproven, although plausible mechanisms of ion channel regulation and chemical defense are often invoked. Molecular approaches and the development of improved detection methods will yield deeper understanding of their physiological and ecological roles. This knowledge will facilitate their further biotechnological exploitation and point the way towards development of pharmaceuticals and therapeutic applications.

## 1. Introduction

Many living forms have developed complex neurological systems to receive and transduce vital information from the environment where they live and to elicit appropriate behavioral responses to such stimuli. Generation of neuro-electrical signals is crucial not only for sensory functions including transmission and processing of information in the neurological center (or brain), but also for muscle contraction, secretion of hormones and distributing response signals to the rest of the tissues. All these electrical signals are conducted by members of the ion channel protein superfamily, comprising more than 140 structurally related pore-forming proteins [1].

Voltage-gated ion channels are the target for a wide range of naturally occurring toxins, including guanidinium and secondary amine analogs and various polypeptide and protein neurotoxins. Among the voltage-gated ion channels, the voltage-gated sodium channel (Na_V_) family was the first to be discovered. These associated proteins are thus considered the founding members of the ion channel superfamily [2]. The Na_V_ pore proteins allow the rapid influx of Na^+^ ions across the cell membrane, typically with a compensatory efflux of K^+^ ions via the respective ion channel. Establishment and collapse of the electrochemical charge gradient across cell membranes generates an ionic imbalance responsible for the initiation of action potentials in nerve, muscle and endocrine cells of animals [3].

The guanidinium neurotoxins, namely saxitoxin (STX), tetrodotoxin (TTX), and their numerous analogs, are naturally occurring alkaloids with a high affinity for binding to Na_V_ channels, thus blocking the influx of Na^+^ ions into the cell. This blockage inhibits the propagation of action potentials in excitable membranes, and this impediment causes effective neuro-muscular paralysis. These toxins have been intensively studied because of their effects on human health after consumption of toxin-contaminated seafood, and morbidity and mortality of primarily marine vertebrate species of fish, mammals and seabirds. More recent research efforts have focused on determining their critical eco-evolutionary roles in the chemical ecology and species interactions in natural marine, freshwater and terrestrial ecosystems.

Toxic events putatively caused by STX and TTX from natural sources have been well documented throughout recorded history and are part of the folkloric traditional knowledge of many indigenous populations. Ancient societies including the Egyptian, Greek, Chinese and Mayan civilizations knew about the toxic properties of certain puffer fishes, presumably containing TTX based upon the characteristic symptomology [4,5]. The use of puffer fish extracts as a key ingredient in zombification preparations for voodoo rituals has been widely known for centuries in the Caribbean [5]. Among certain native populations of the Pacific northwest of North America, wild shellfish consumption is traditionally avoided during periods of “shiny water”, or bioluminescence caused by certain dinoflagellates. Blooms of the STX-producing dinoflagellate *Alexandrium catenella* have been recognized as a major contributor to natural surface ocean bioluminescence and simultaneously to high shellfish toxicity in the northwest Pacific region for many decades [6]. Within the last 50 years, such dinoflagellate blooms known to produce to STX analogs have apparently expanded in biogeographical range, and have contributed to increased magnitude and frequency of toxic events around the world.

## 2. Origin and Proximal Sources of Guanidinium Toxins

**Figure 1:**
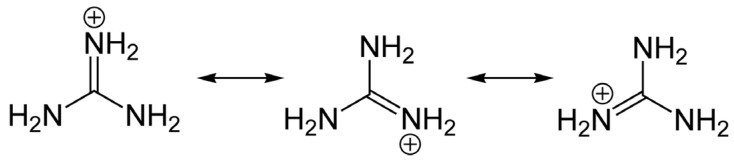
Structure and ionization of the guanidinium group. Saxitoxin (STX) has two guanidinium moieties and therefore two dissociation constants: pK_a_ 8.22 for the 7,8,9 guanidinium group and 11.28 for the 1,2,3 group [12]. Tetrodotoxin (TTX) has only one guanidinium moiety, with a pK_a_ of 8.76. These groups are protonated under physiological conditions, with a ^+^1 charge [13].

Toxin analogs belonging to the STX and TTX families share common guanidinium moieties (Figure 1), which accounts for their neurotoxicity and similar molecular targets, but these toxin groups differ widely in organismal origin and biogeography. The distribution of TTX and its analogs is highly diverse, as these toxins can be found in aquatic and also in terrestrial environments. Human intoxications by TTX are most often linked with the consumption of certain puffer fish species from the marine environment, particularly in tropical and sub-tropical regions; hence the syndrome is often referred to as “puffer fish poisoning” (PFP) [7]. Nevertheless, recent detection of TTX in gastropods [8,9] and in bivalve mollusks from Europe [10,11] at levels of concern for human consumers of seafood, suggests that the risk may be more widespread than formerly assumed.

For a long time it was believed that TTX was produced exclusively by fishes of the family Tetraodontidae, but now this toxin and its analogs are known to occur in a wide diversity of often phylogenetically unrelated organisms [14] of either terrestrial or marine origin. In addition to bacteria, these include species of newts, crabs and frogs, as well as some gastropods, bivalve mollusks, sea slugs, star fishes, blue-ringed octopus and ribbon worms [15,16,17,18,19,20,21,22]. During most of the 20th century there was a raging debate regarding the origin of TTX in marine and terrestrial fauna—endogenously produced by metazoa or by epi- or endo-symbiotic bacteria or exclusively by free-living bacteria harbored briefly during gut passage of ingested food. Now it is known that most (perhaps all) marine metazoa do not themselves produce these toxins, but rather they are synthesized by different genera of bacteria [22,23]. In the marine environment, TTX-producing bacteria most frequently belong to species of *Actinomyces*, *Aeromonas*, *Alteromonas*, *Bacillus*, *Pseudomonas* and *Vibrio*, among other common genera [24]. Marine fauna acquire the toxin-producing bacteria via the food web, whereby the bacteria can persist in their guts, or by parasitism or symbiosis, with the bacteria lodged within or on their skin [24,25,26]. In these cases, the metazoa merely serve as vectors or hosts for the toxigenic bacteria. A few studies have suggested an endogenous origin of TTX for certain puffer fish species, such as *Takifugu* (=*Fugu*) *niphobles* [27], but this is considered a rare case and requires further confirmation. Knowledge of the origin and distribution of TTX among terrestrial toxic organisms is rather different; to date no TTX-producing bacteria have been isolated from any amphibian species that possesses this toxin [17,28]. This has led to the hypothesis that TTX production in these organisms is endogenous as a defense mechanism, and that extant bacteria are no longer involved in their biosynthesis, although the biosynthetic genes may have been originally bacterial. Curiously, TTX appears to be essentially absent from fauna living exclusively in freshwater habitats, and not just returning to aqueous systems for breeding.

Saxitoxin and its analogs are alternatively known as “paralytic shellfish toxins” (PSTs) because they cause the syndrome known as “paralytic shellfish poisoning” (PSP) in human consumers of toxin-contaminated seafood. The PSTs accumulate in many marine species via the food web, particularly in bivalve shellfish by suspension-feeding on toxic dinoflagellates, but also in crustaceans and gastropods. The toxins may be transferred to marine mammals and sea birds that feed by diverse mechanisms on zooplankton, mollusks, ichthyoplankton and fish. In the sea, PSTs are produced by members of three genera of free-living marine dinoflagellates associated with harmful algal blooms (HABs): these include about a dozen species of *Alexandrium*, and a single species each of *Gymnodinium* (*G*. *catenatum*), and *Pyrodinium* (*P. bahamense* var. *bahamense*) [29,30]. According to a review by Hallegraeff [31], close to 2000 PSP cases are reported yearly around the world, with a mean fatality rate of about 15%. Children are known to be more susceptible and suffer a higher fatality rate [32], which is only partially attributable to their generally lower body mass.

Many species of brackish and freshwater filamentous cyanobacteria [28,31,32,33] from the orders Oscillatoriales and Nostocales are also capable of producing STX and analogs, but they are phylogenetically unrelated to the toxigenic dinoflagellates in the ocean and tend to occupy different habitats. Various strains of species within the cyanobacterial genera *Cylindrospermopsis*, *Dolichospermum* (previously *Anabaena* [33]), *Aphanizomenon*, *Planktothrix* and *Lyngbya* [34,35] are known to biosynthesize PSTs, even in the absence of other associated bacteria. Although no acute human PSP events have been recorded from freshwater systems, e.g., from consumption of fish or crustaceans, there have been cases of livestock poisoning by drinking from freshwater bodies during high magnitude cyanobacterial blooms containing PSTs [36]. The presence of these toxins is also of concern regarding drinking water supplies for humans and for wild animals. Recreational water resources can be also affected by the presence of cyanobacterial blooms containing PSTs [37]. There are many known cases of contact toxicity and skin irritation linked to cyanobacterial blooms, but because some cyanobacteria produce multiple toxins the exact role of PSTs in these syndromes has not been established. There is little research on contact expose to PSTs released from algal blooms and it is yet unknown if these toxins are effectively transferred to humans via this route.

## 3. General Chemical and Toxicological Properties of Guanidinium Toxins

Toxin molecules from the STX and TTX groups share a number of chemical properties that account for their toxicity. They are heat stable alkaloids, and thus cooking or boiling food bearing the toxins will not generally lead to sufficient loss or deactivation of the toxins to prevent poisoning the consumer. The PSTs are subject to thermal decomposition under alkaline conditions, but this tends to render the food inedible. These toxins are rather water-soluble and therefore some toxin will be extracted during cooking in liquid; if the liquid fraction is then discarded, the net toxicity of the food will be somewhat reduced. This is, however, not a reliable method of toxicity reduction and in fact heating, especially under mild acidic conditions, tends to convert the PSTs to more toxic analogs.

The guanidinium toxins are small molecules with low molecular weight (typically between 200 and 600 Da) and are not known to form macromolecular complexes. Compared to other Na_V_ channel blockers, such as the µ-conotoxin group of polypeptide toxins from marine mollusks, it is amazing how these tiny structures, formed by less than twenty carbon and three to five nitrogen atoms, plus hydrogen and oxygen, are capable of blocking the Na_V_ channels with such high affinity to cause neurological symptoms that can lead to death in severe cases of exposure.

**Figure 2:**
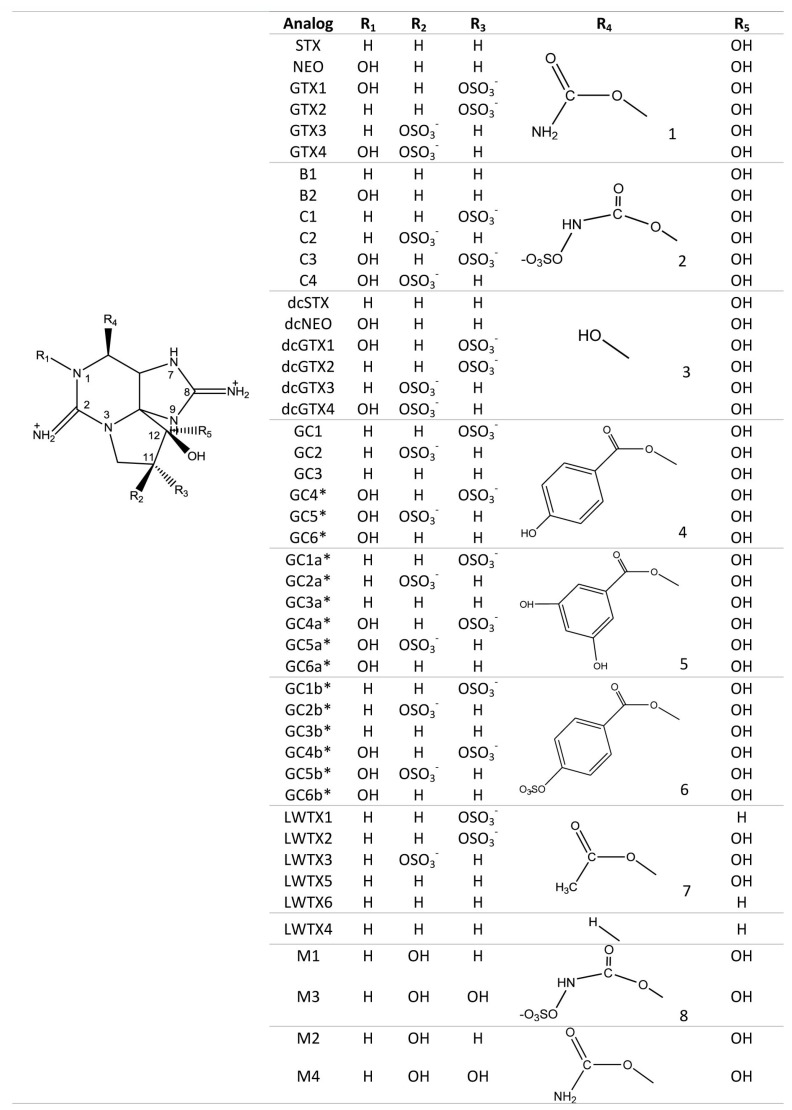
Saxitoxin and major naturally-occurring analogs produced among marine dinoflagellates, various freshwater and brackish water cyanobacteria (e.g., LWTX1-LWTX6 from *Lyngbya*) and metabolic transformation products (e.g., M-toxins) found in mussels (*Mytilus*). Left: the 3,4,6-trialkyl tetrahydropurine skeleton with two guanidinium groups, common to all STX analogs. STX, saxitoxin; NEO, neosaxitoxin; GTX1-4, gonyautoxins 1 to 4; B1, B2, toxins B1 and B2; C1-C4, toxins C1 to C4; dcSTX, decarbamoyl saxitoxin; dcNEO, decarbamoyl neosaxitoxin, dcGTX1-4, decarbamoyl gonyautoxins 1 to 4; LWTX1-6, lyngbyatoxins 1 to 6; M1-M4, *Mytilus* toxins 1 to 4. Asterisks* for the GC-toxins refer to putative structures determined primarily by LC-MS/MS, with NMR support in some cases, but which remain to be confirmed.

Members of the STX group bear two guanidinium groups, whereas TTXs bear only one (Figure 1). This chemical characteristic confers either one or two positive charges to the molecules at physiological pH, which has important implications for their toxicity [38]. To date, the STX group is represented by more than 50 natural analogs, comprising the PSTs synthesized and metabolized by dinoflagellates and cyanobacteria, plus analogs created via biotransformation in other species [37] (Figure 2).

**Figure 3:**
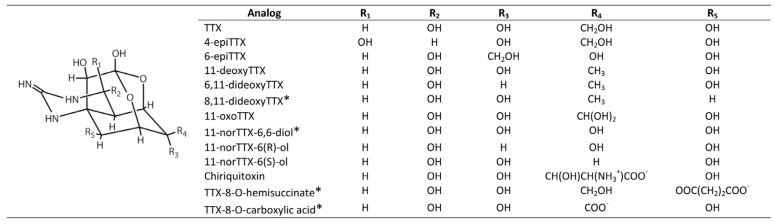
Tetrodotoxin (TTX) and analogs. The asterisks* indicate synthetic analogs, whereas those without asterisks refer to major naturally occurring compounds.

The TTX group comprises more than 25 natural analogs found among marine and terrestrial fauna and associated bacteria, plus synthetic analogs [24] (Figure 3).

Synthetic analogs of both toxin groups have been produced to study the biosynthetic pathways and to explore specific toxicity and potential therapeutic applications. In any case, the discovery of new natural guanidinium toxins has not been exhausted; the major analogs of human health significance have likely been identified and structurally characterized but further metabolites of unknown toxicity will undoubtedly be uncovered.

Guanidinium toxins are considered amongst the most potent known natural toxins, with lethal dosages for humans in the low milligram range for the most toxic analogs of TTX [22] and STX [37] (Table 1 and Table 2).

As the only marine natural product declared a chemical weapon [49], STX has been placed on Schedule 1 of the Chemical Weapons Convention [50]. Toxin potency in mammalian systems varies with structural differences among analogs from both STX and TTX group [38]. Among the STX group, the carbamoyl derivatives are typically the most potent, with decarbamoyl analogs of intermediate potency and N-sulfocarbamoyl analogs much less toxic. The deoxy analogs of TTX are typically less toxic than TTX, whereas the hydroxyl analogs, acting as hydrogen bond donors, are more toxic because of enhanced binding to Na_V_ channels [24]. Extensive toxicity studies have not been conducted with all the natural analogs of either guanidinium toxin group, mainly due to the lack of sufficient purified toxins. Nevertheless, in cases where empirical toxicological data are not available, their structures provide information to generate hypotheses about their potency based upon binding simulations [39].

## 4. Symptomology and Etiology of Exposure to Guanidinium Toxins

At the physiological level, there is little to distinguish the effects of TTX versus STX poisoning in humans or among other higher vertebrates with comparable neuro-muscular systems, other than the fact that with PSP as opposed to TTX poisoning there is rarely significant hypotension. In humans, guanidinium toxin poisoning elicits a series of distinctive symptoms: around 30 min after consumption of the toxic food (and depending on the dosage), the victim begins to experience a characteristic sequence of paresthesias: tingling of tongue and lips, and sometimes in other tissues if the victim has been in contact with the toxin near an open wound (even a superficial cut). These tingling symptoms can be followed by a sensation of floating, headache, vomiting, muscle weakness and a lack of muscle control or coordination of voluntary movements (ataxia) that can cause difficulties with speech, eye movement and swallowing. These sensations and effects may reach extremities, even fingers and toes. After a while (typically a few hours), paralysis starts, and voluntary movements are lost, but consciousness remains unaffected. With high doses, death may occur due to paralysis of the muscles involved in respiration, such as the diaphragm [43,51]. The lethal oral dose in humans is approximately 1 to 4 mg STX depending upon the age, individual susceptibility and physical condition of the victim.

Analysis of tissue and body fluids from fatal PSP victims have shown distribution of the toxins in brain, bile, cerebrospinal fluid, liver, spleen, heart, thyroid and adrenal glands, kidneys, pancreas and lungs [52]. This systemic distribution is primarily due to the high mobility and hydrophilic nature of the toxins and explains the neurological and gastrointestinal symptoms. The guanidinium toxins are capable of crossing the blood-brain barrier, at least in high doses [53].

To date there is no known targeted antidote for acute guanidinium toxin poisoning. Anticurare drugs are known not be effective, whereas administration of anticholinesterase agents previously tried as therapeutants, such as the nonselective muscarinic acetylcholinergic antagonist atropine, can actually be counter-productive. The ineffective or counterproductive role of anti-cholinesterase agents in TTX or STX poisoning remains poorly defined but may be due to the fact that the toxin blockade leading to paralysis is not antagonized by anticholinesterase agents. At least in the case of TTX poisoning, neuromuscular transmission is interrupted at the motor axon and muscle membrane and not at the end plates (cited in [54]). The stimulant DL amphetamine (benzedrine) may, however, provide supportive aid during artificial respiration and in reducing the recovery period. The poisoning victim requires immediate medical attention, often with respiratory assistance. In some cases, oral administration of activated charcoal to capture remaining toxin that has not yet been taken up from the victim’s digestive system and/or gastric lavage with antacid agents such bicarbonate can aid in mitigating symptoms. Residual symptoms can remain for up to a week, but no long-term clinical effects have been reported [49,55].

Fatalities after acute exposure to PSTs are rather uncommon in countries with a well-administered toxin surveillance program for seafood, but low dosage extended exposure to the toxins may frequently occur in communities that depend primarily on seafood for subsistence. The annual intake of potentially toxin-contaminated seafood in these indigenous communities is by far greater than average, and natives may chronically consume untested shellfish containing low toxin doses. The claim that PST tolerance can occur [56] may indicate that individuals frequently and chronically exposed to sub-lethal doses of the toxins could ingest amounts higher than safety guidelines with no deleterious symptoms [53], although controlled epidemiological studies are lacking to confirm this response. In any case, the mechanism of such putative toxin resistance is not understood, but is unlikely to be immunogenic because of the small molecular size and non-proteinaceous nature of the PSTs.

Although subtle low dosage adverse effects may occur, there is little research on guanidinium toxin effects at low-chronic doses in humans. Studies on rat cortical neurons showed that chronic exposure to TTX cause dendrite retraction, loss of dendritic spines and degeneration of the neurons within a period of 1–2 weeks, as well as apoptotic processes triggered by miniature excitatory postsynaptic currents [57]. Experimental work on neurites indicated that long-term exposure to low doses of guanidinium toxins could affect neurogenesis during CNS development [58].

Apparently, chronic exposure to guanidinium toxins does not exclusively affect the nervous system. The possibility that these toxins could have adverse effects on other physiological systems is supported by the observed reduction in metabolic enzyme activity in different animal models; there is also the potential for enzyme polymorphisms that could yield inter-individual differences in response to STX [53]. In some wild fish, adverse effects such as epithelial hyperplasia in the gills and liver necrosis have been observed after extended exposure to low doses of PSTs [59]. Fish and mammalian in vivo models have shown both significant changes in antioxidant mechanisms and DNA damage in response to these toxins [60,61]. Antioxidant enzymes were significantly reduced in the liver of mice exposed to low (sub-lethal) concentrations of the epimeric pair GTX 2/3; a similar effect was also noted when the less toxic analogs C1/2 were administered [60]. Drinking water spiked with STX and provided to laboratory rats for 30 days induced changes in the antioxidant mechanisms in brain and liver [62]. These changes could cause an increase in oxidative stress [63], although how PSTs could directly or indirectly mediate these reactions is still unknown. All these results point to the possibility that low-dose exposure to guanidinium toxins, even when unnoticed, could still have critical long term physiological consequences, and not only within the nervous system.

## 5. Mode of Action and Ion Channel Targets

Sodium ion channels are molecular targets for many marine neurotoxins, but the binding sites, relative affinities and toxic potency are specific for the respective pharmacophore and hence elicit widely different pharmacological responses. The potent dinoflagellate polyketide palytoxin, for example, targets the Na^+^:K^+^ ATPase [64], which is not a voltage-gated channel, whereas STX can interact with voltage-gated Na^+^, Ca^2+^ and K^+^ ion channels to a greater or lesser extent, with potency determined by the structure and polarity of the individual analogs. Interaction of STX with the human ether-á-go-go (hERG) potassium channel has been reported [65], with the mechanism of inhibition of the channel by STX described as completely distinct from the way in which it inhibits Na_V_ channels. The hERG K^+^ channels are inhibited by modifying channel gating rather than by blockage of the pore, and not one, but four or more toxin molecules are able to bind to these channels [65,66]. The action of STX on calcium channels has also been proven. The effect of STX on L-type calcium channels (L-Type *I_Ca_*) is via the reduction the Ca^2+^ currents for this type of channel in ventricular myocytes, but this effect has not been observed with TTX; it is possible that these channels possess a binding site for STX but not for TTX [67].

In any case, the guanidinium moieties (Figure 1) on STX and TTX analogs are the pharmacophores responsible for the blocking activity on the Na_V_ channels. These guanidinium groups, comprising a central carbon atom and three nitrogen atoms with a positive charge at physiological pH, confer the binding capacity to Site 1 of the Na_V_, thereby partially or completely blocking the inward Na^+^ ion current. The guanidinium toxins are known to elicit a multiplicity of effects on various ion channel types, but their primary toxic effects are mediated via voltage-gated ion channel blockage, specifically by binding to the Na_V_ channels of excitable cells, mainly in muscles and nerves. Blockage of Na^+^ ion entry affects the sensory systems, muscles and nerves of most animals, but particularly vertebrates [68] with highly sophisticated and differentiated neurological systems.

Prior to empirical knowledge of the mode of action of guanidinium toxins, ion channels were essentially unknown. The discovery that these toxins interfered with the generation of action potentials in nerves and voluntary muscles with high potency [69] led to numerous investigations on the electrophysiological mechanisms and structures involved in the generation of action potentials. Application of a voltage-clamp technique with lobster axon preparations showed a specific potent action of TTX in preventing the increase of Na^+^ ion conductance associated with the so-called, “sodium carrying mechanism”. This early pioneering study by Narahashi and co-workers [70] initiated further studies on what later would be called ion channels. Soon thereafter it was noted that both guanidinium toxins (STX and TTX) shared similarities in mode of action, by interfering with the production of action potentials in nerves and skeletal muscle at nanomolar concentrations. These toxins became useful additions to the arsenal of pharmacological tools in the study of the molecular structure of excitable membranes [71]. Later studies confirmed that the positive charges of the guanidinium groups were substantially responsible for elicited toxicity, and this led to the discovery of the ion channel families.

The guanidinium groups fit the external orifice of the Na_V_ channels but the toxin molecule is too large to penetrate the pore, resulting in the clogging of the ion passage [70,72]. Channel isoforms are now classified in terms of “sensitive” or “resistant”, depending on the binding affinity of TTX [73] (Table 3). This sensitivity property is the result of point amino acid substitutions in the ion channel structure but is only beginning to be understood in the context of evolutionary origin and physiological significance [69].

Some predators that fed on highly toxic prey, such as newts of the genus *Taricha*, which are known effective TTX producers, have evolved resistance to high levels of the toxin. For example, certain amino acid sites have been identified in the Na_V_1.4, 1.6 and 1.7 that confer resistance to TTX for the garter snake *Thamnophis sirtalis*, a newt predator. These channel isoforms are found in the muscle as well as in the peripheral nervous system, where they can be exposed to the ingested toxin, and thereby they have evolved resistance. Central nervous system ion channels, such as Na_V_1.1–1.3, have not shown any resistance to TTX, and this is consistent with protection from toxin exposure by the blood-brain barrier [74].

**Figure 4:**
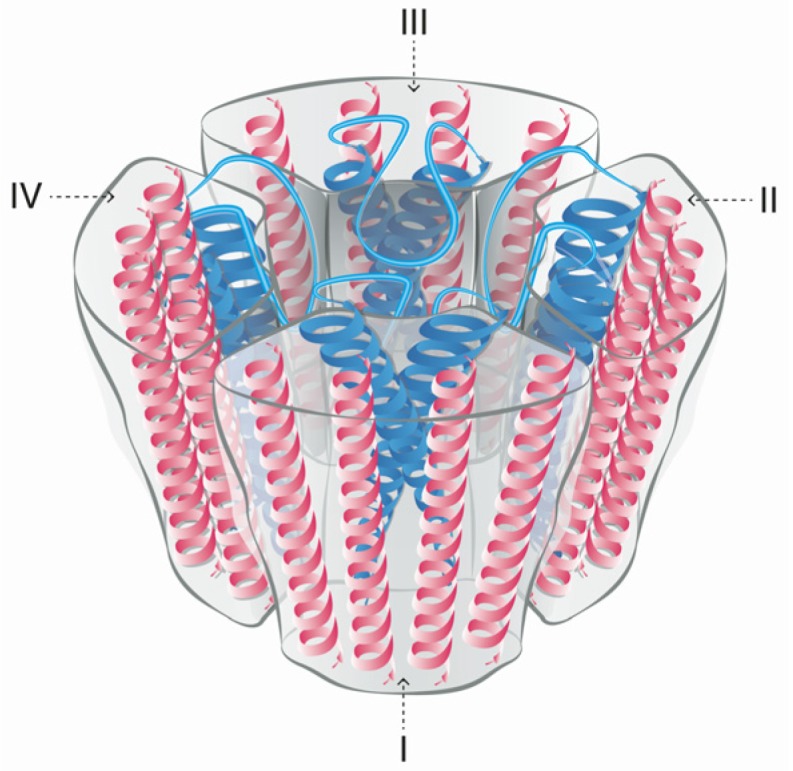
Schematic figurative representation of a Na_V_ channel indicating the four α-subunit domains (I–IV). Each domain has six transmembrane segments S1–S6 (shown as protein spirals). The pore-forming segments (S5–S6) in each domain are indicated in blue.

**Figure 5:**
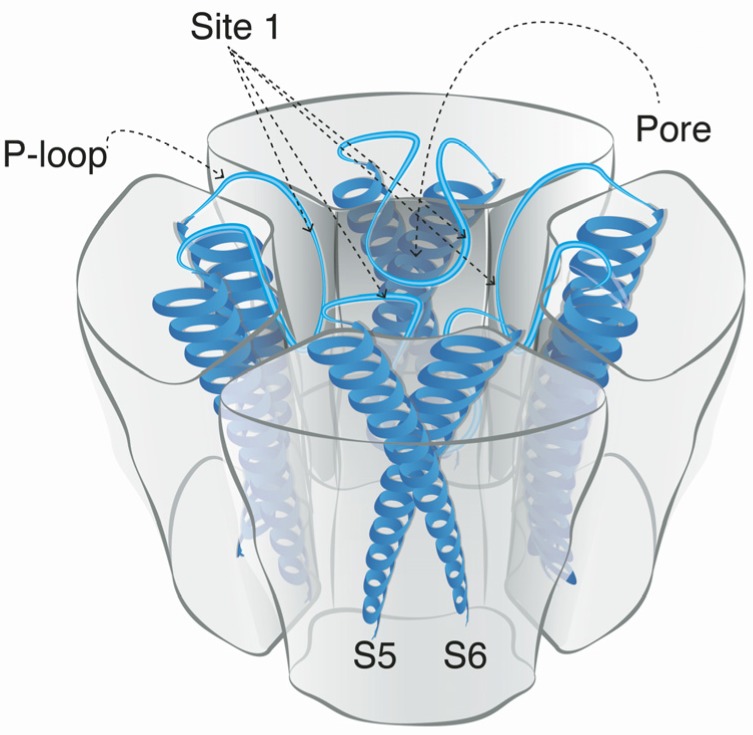
Schematic figurative representation of the α-subunit of the Na_V_ channel. The protein structure shows the location of Site 1, the binding site for STX and TTX and their respective analogs.

Voltage-gated sodium channels are pore-forming membrane proteins that consist of a single protein complex that forms one α- subunit of 220–260 kDa and one to three auxiliary β-subunits of approximately 33–36 kDa [3,76]. The α-subunit comprises four homologous domains (I–IV), that are thought to form a circumference around the ion-conducting pore. Each domain contains six hydrophobic transmembrane segments (S1–S6) (Figure 4). Two of these segments (S5 and S6) form the internal portion of the pore, and have a membrane-reentrant loop between them, called the P-loop. These P-loops form the fine extracellular end of the pore (Figure 5). The β-subunits are small proteins that interact with the α-subunits, altering their physiological properties and their localization, but they do not have an active relationship with the ion influx. These subunits are considered to be a recent evolutionary addition to the pore-forming proteins and have only been identified in vertebrates [3,77,78].

To date, nine Na_V_ α-subunits have been fully functionally characterized and designated: Na_V_1.1 to 1.9, comprising a single family of proteins [2]. The α-subunits differ in terms of tissue distribution in adults and developing organisms, pharmacological and electrophysiological properties, and response to nerve injuries [79]. All cells containing Na_V_ channels co-express multiple isoforms [80].

The Na_V_ channels have a selectivity filter that allows the entry of Na^+^ ions and inhibits the entrance of other positively charged molecules/ions. This filter consists of one amino acid from each of the four domains (I–IV), respectively: Asp (D)–Glu (E)–Lys (K)–Ala (A) (called the DEKA motif) [39]; this DEKA motif comprises one residue from each of the four P-loop regions. Mutational and binding mapping studies led to the identification of the four main residues in the selectivity region located on the outer portion of the pore [81,82,83,84,85]. Three positions from the DEKA motif there is a ring of negatively charged amino acids (Glu (E)–Glu (E)–Met (M)–Asp (A) in mammals) that creates an electrostatic cloud to attract the Na^+^ ions into the pore [86]. Any alteration of the amino acids forming the DEKA moiety of the selectivity filter, or in this charged ring, can reduce the Na^+^ ion conductance across the pore [87].

The binding site for guanidinium toxins is located in the α-subunit, specifically in the outer vestibule, a water-filled region formed by the four P-loops [82]. This is the binding Site 1, which is blocked specifically by guanidinium toxins (Figure 5). The affinity of these toxins in the Na_V_ channel is 1:1; this means that one molecule binds to one ion channel, blocking it completely or partially [81,88].

**Figure 6:**
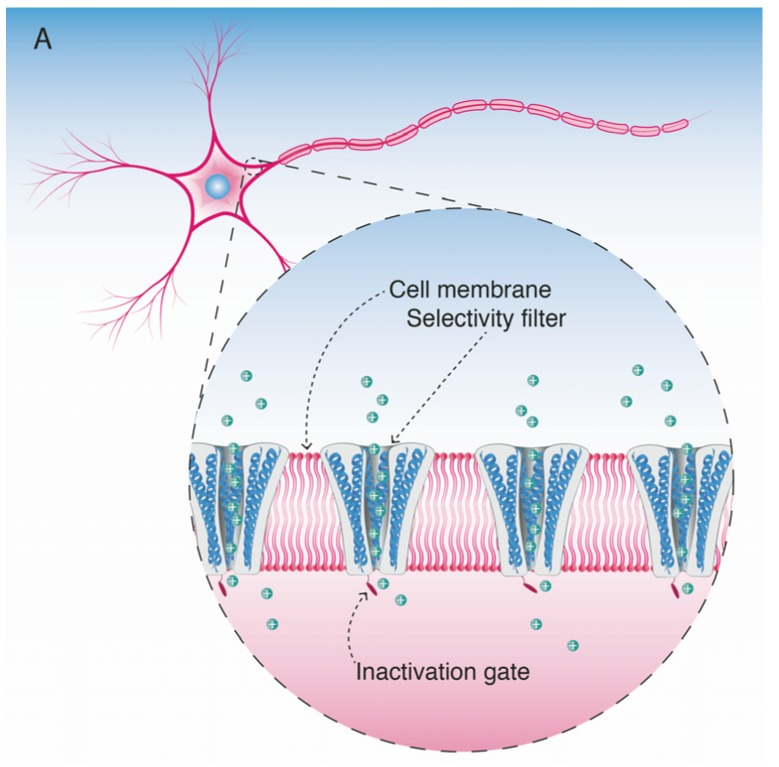

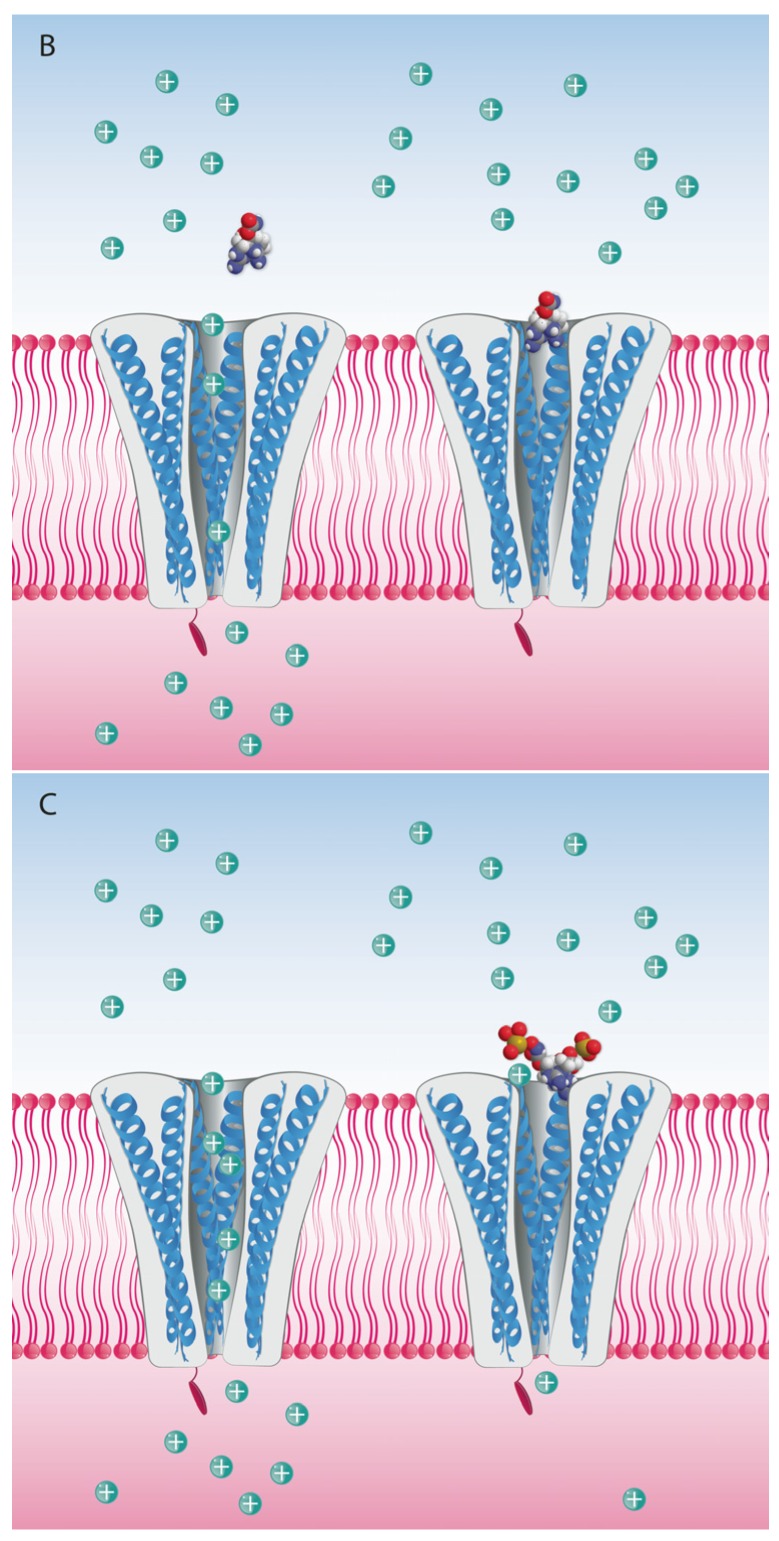
Schematic figurative representation of the Na_V_ channels in a neuronal cell membrane. (**A**) Normal Na^+^ ion flux through the pores; (**B**) Na_V_ channels blocked by STX molecules; (**C**) Na_V_ channels partially blocked by N-sulfocarbamoyl C1 toxin. Here the steric hindrance effect and negative charge of the N-sulfo-groups are hampering the complete blockage of all pores, still allowing some Na^+^ ions to pass through the non-blocked pores.

Different analogs are considered to bind to the same site in the pore, but with different affinities depending on the functional groups present for each analog. One hypothesis for potency differences among the STX analogs is that they are due to steric hindrance effects, whereby larger functional groups such as sulfate or benzoyl moieties interfere with the blocking capacity (Figure 6). Nevertheless, this has not been proven, and there are alternative hypotheses about differences in the mode of binding of TTX versus STX and their respective analogs [89]. An alternative, but not mutually exclusive, hypothesis is that lesser potency is due to the compensatory reduction of the net positive charges, and consequent reduced affinity for the Site 1 when the molecule has functional groups with negative charges that balance the net charge [39], e.g., in the case of the N-sulfocarbamoyl C-toxins.

Recent studies [89] have shown that there are unexpected differences in affinity for STX analogs and TTX among certain Na_V_ isoforms. Walker et al. [89] demonstrated that whereas rat Na_V_1.4 and human Na_V_1.7 showed comparable affinity to TTX, their affinity to STX exhibited a 250-fold difference between both isoforms. In comparative analyses of the channel amino acid sequences they identified two amino acid variations in the α-subunit, and this variation was unique for Na_V_1.7 in humans and other primates. Through electrophysiology experiments with mutant forms of both channel isoforms, they proved that specific amino acid substitutions are important modulators of STX affinity to Na_V_. These results are a challenge to the conventional belief that both guanidinium toxin groups have comparable activities for all channel sensitive isoforms, and demonstrate the need for deeper research to fully understand the interactions between guanidinium toxins and Na_V_ channels.

## 6. Evolution of Voltage-Gated Na^+^ Ion Channels and Guanidinium Toxin Genes

The evolutionary history of Na_V_ ion channels and the phylogenetic origin of the guanidinium toxins pose challenging questions for understanding the functional role of these toxins, particularly with respect to vertebrate nervous systems. Molecular genetic evidence indicates that the evolution of Na_V_ channels predates the origin of the nervous system present in all animals, except for Porifera (sponges) and primitive basal proto-metazoans (i.e., the putative eumetazoan sister group Placozoa). Voltage dependent calcium (Cav) channels apparently developed even earlier in unicellular eukaryotes (Protista), where they play a critical role in intracellular signaling pathways, as for multicellular organisms. The Cav and Na_V_ channels share key features, such as the presence of four domains, each of which has a pore loop. The correlating hypothesis is that Na_V_ channels were derived from Cav channels at the origin of the nervous system [90], thereby conferring the ability to conduct action potentials without interfering with intracellular calcium fluxes. This hypothesis is also consistent with the apparent lack of sodium ion currents in sponges [91]. Empirical support for this hypothesis and unique insights into the evolution of voltage-gated ion-channel genes was provided by in silico genomic and gene expression sequence analysis of ion channel protein genes of basal animals and their close unicellular relatives, including fungi—essentially confirming this evolutionary relationship [92].

The evolution of Na_V_ ion channels in vertebrates involves increasing complexity and refinements in neuromuscular coordination, and hence more sensitivity and susceptibility to interference with the generation of action potentials by guanidinium toxins. Adaptive sequence evolution in Na_V_ channels reveals patterns of increasing complexity, specialization and diversity (reviewed by [93]), particularly among vertebrates. The primary targets of guanidinium toxins are Na_V_ channels, at least in term of relative potency, but STX may also act on Ca^2+^- and human hERG K^+^ channels (cited in [53]), although in the latter case by modifying K^+^ channel gating rather than by blocking the channel. Neuromuscular responses in invertebrates tend to be more Ca^2+^- ion channel dependent, and they possess alternative channels (both Na_V_1 and Na_V_2) rather than the exclusive Na_V_1 family in vertebrates [93]. For these reasons, and the consequent relative insensitivity to guanidinium toxins, bivalve mollusks and certain other invertebrates can sequester a body burden of PSTs that would prove lethal to vertebrates, often with little apparent physiological effect. The response to guanidinium toxins mediated by Na_V_ ion channel interference is highly species-specific, but conduction of action potentials in the nerves of certain bivalve mollusks persists even after prolonged exposure to high concentrations of TTX or STX [94]. Nevertheless, deleterious effects, e.g., inhibition of burrowing behavior and siphon retraction in the soft-shell clam *Mya arenaria* [95], can be elicited in some cases. In fact, adaptive evolution of toxin resistance in populations of *M. arenaria* chronically exposed to blooms of PST-producing dinoflagellates is evidenced by mutation of the Na_V_ channel protein via a single amino acid substitution from Glu to Asp in the outer pore loop [95], thereby conferring a 1000-fold decrease in toxin affinity to the binding site.

The evolutionary history of the vertebrate Na_V_ is a fascinating story which can only be briefly referenced here, with respect to evolution of TTX resistance. Essentially, this first involves extensive channel gene duplication including Na_V_1 and selective retention in teleost fish and terapods [85]. The complexity of multiple Na_V_ channel genes in vertebrates poses a fundamental question as to the development of TTX resistance (against auto-toxicity) in amphibians (frogs and newts) and toxigenic fish, such as *Fugu* and other puffer fishes. The leading hypothesis contends the gradual accumulation of resistance via adaptive gene mutations in the Na_V_ channels [85]. Predator-prey interactions between sympatric versus non-sympatric garter snakes and toxigenic newts are also suggestive of parallel evolution of TTX-resistance in independent populations and species of resistant snakes. Sequences of expressed Na_V_1.4 channels encoded by the *scn4a* gene reveal corresponding amino acid substitutions in the TTX binding site pore [96].

In the context of ion-channel interactions with guanidinium toxins it is instructive to consider the probable evolutionary origins of the biosynthetic genes in cyanobacteria and dinoflagellates. Although current speculation is that the biosynthetic genes for STX arose first in bacteria, there is no conclusive evidence that extant bacteria other than certain cyanobacterial species are capable of synthesizing these toxins. The first putative STX biosynthetic gene cluster (*sxt*) was identified in the cyanobacterium *Cylindrospermopsis raciborskii* T3 [97] and minor gene cluster variants were later confirmed in other cyanobacteria, including *Anabaena circinalis* AWQC131C and *Aphanizomenon* sp. HN-5 [98,99]. A core set of 14 genes (*sxtA-sxtI*, *sxtP-sxtR*, *sxtS* and *sxtU*) were originally found among PST-producing cyanobacteria, and about a dozen more variable elements of the *sxt* gene cluster have been subsequently identified. Presence or absence of individual elements of the gene cluster can account for the toxin composition profile differences among strains and species [100].

Comparative phylogenomic analysis of draft genomic assemblies of putative *sxt* genes in various filamentous cyanobacteria [101] suggests the biosynthetic genes were derived via multiple horizontal gene transfer (HGT) events from alternative origins, including precursor bacteria, and accompanied by subsequent coordination within and among multiple cyanobacterial lineages. Under this scenario, the establishment of ancestral toxigenic strains within filamentous cyanobacteria was succeeded by widespread and common loss of the relevant genes, and hence the patchy distribution of toxigenicity among extant cyanobacterial lineages.

This phylogenomic evidence from toxigenic cyanobacteria leaves open the questions as to whether or not the development of STX gene homologs in dinoflagellates arose via single or multiple HGT events directly from STX-producing cyanobacteria or independently, perhaps from bacterial precursors. Close homologs of the cyanobacterial genes were later identified as nuclear-encoded genes in the PST-producing dinoflagellates *Alexandrium fundyense* (now renamed *A. catenella*) and *A. minutum* [102]. This similarity led to the hypothesis that the biosynthetic genes arose via horizontal gene transfer (HGT) between ancestral STX-producing bacteria and dinoflagellate nuclei. Nevertheless, comparative analysis of the structure and assembly of respective STX gene clusters in cyanobacteria and dinoflagellates indicates enough dissimilarity to suggest some independent evolution. The fact that among dinoflagellates, PSTs are produced by two genera within one family (*Alexandrium* and *Pyrodinium*) and by a single species from a distant dinoflagellate order (*Gymnodinium*) does tend to indicate that such bacteria-to-dinoflagellate horizontal gene transfer would likely have taken place prior to the separation of the gonyaulacoid genera *Alexandrium* and *Pyrodinium*. Under this scenario, this event was followed by a dinoflagellate-to-dinoflagellate horizontal gene transfer into *G. catenatum* [102].

Whether or not the evolution of Na_V_ ion channels reflects the functional role of guanidinium toxins and the evolution and maintenance of the corresponding biosynthetic genes in cyanobacteria and dinoflagellates remains unknown. The origin of filamentous bacteria dates back to at least 2800 Ma, and molecular genetic evidence proposes the emergence of the *sxt* gene cluster by before 2100 Ma, early in the divergence of the Nostocales [103]. The proposed adaptive advantage of STX analogs in mediating Na^+^ ion regulation and transport in extant toxigenic cyanobacteria from fresh and brackish waters [104,105] is consistent with this functional role. Indeed is it conceivable that the pre-Cambrian precursor to Na_V_ channels-primitive voltage dependent K^+^ (K_v_) ion channels—may have been the ancestral target for the guanidinium toxins [103]. Potassium leak and K_v_ ion channels originated 3000 Ma in bacteria [93] and are present in all prokaryotes and eukaryotes. Under this interpretation, the osmoregulatory effect on Na^+^/K^+^-ATPases and proton pump H^+^-ATPases (reviewed in [106]), for regulation of salinity/cell volume and pH equilibrium, respectively, could have been crucial to the co-evolutionary development of biosynthetic genes for PSTs and susceptible ion-channels in cyanobacterial lineages.

The evolutionary history of the toxigenic dinoflagellates, as for other eukaryotes, is rather different from cyanobacteria and involves many unresolved complexities, not least of which involves the shared elements of the *sxt* gene cluster. The extensive fossil record of dinoflagellates extends back at least 245 Ma to include the Mesozoic and Cenozoic eras, but molecular phylogenetic analysis of modern dinoflagellates and morphological evidence (e.g., probable homology with acritarchs) suggests an even earlier Precambrian (>570 Ma) origin [107]. Morphotypes consistent with gonyaulacoid dinoflagellates first appear in the fossil record of the early Mesozoic, but the fossil record of extant dinoflagellates known to produce guanidinium toxins extends only back around 10,000 years. Molecular genetic evidence on the *Alexandrium tamarense* species complex proposes a molecular clock that splits the toxigenic group at about 23 Ma [108]. The fossil record and inferences from phylogenomic analysis, primarily of rDNA genes, tend to suggest an ancient paraphyletic origin for biosynthesis genes for guanidinium toxins in dinoflagellates, but with more recent secondary loss of biosynthetic capacity at the generic and specific level. Current hypotheses on the origin of the biosynthetic genes for PSTs in dinoflagellates-autonomous toxin production by symbiotic bacteria; convergent evolution; single or multiple HGT events—were elegantly presented in a recent comprehensive review [109] and hence the arguments will only be briefly summarized here. Empirical evidence for the production of PSTs by endosymbiotic or externally associated bacteria has been substantially weakened by the failure to confirm the synthesis of these toxins in isolated bacteria and by the confirmed location of the *sxt* gene cluster in the dinoflagellate nuclear genome. The hypothesis that convergent evolution of biosynthetic genes may account for their occurrence in phylogenetically unrelated dinoflagellates and cyanobacteria remains open because of the shared domain structures of the *sxtA* [102] and *sxtG* [110] core genes. Nevertheless, the occurrence of multiple *sxt* homologs with high sequence identity [110,111] renders this convergent origin hypothesis unlikely in such highly phylogenetically unrelated groups that lack a common ancestor. Hence, single- or multiple-HGT of biosynthetic genes to dinoflagellates from Proteobacteria and/or cyanobacteria remain as the most likely scenarios (reviewed in detail by [109]). In fact, half of the known dinoflagellate *sxt* homologs are linked to probable proteobacterial origin [111], although no extant strains are known to biosynthesize these toxins. The collective evidence argues for evolution of the STX biosynthetic genes in cyanobacteria prior to the massive HGT of the initial pathway genes (*sxtA*, *sxtG* and *sxtB*) to the dinoflagellates. In this scenario, the dinoflagellate ancestor of the extant toxigenic gonyaulacoid dinoflagellates *Alexandrium/Pyrodinium* then acquired and assembled the additional genes in the pathway from multiple prokaryotic sources (but not from cyanobacteria). The STX pathway genes were subsequently modified and incorporated into a “eukaryotic structure”, and were subject to loss or acquisition among multiple dinoflagellate lineages to yield a paraphyletic distribution.

Irrespective of the complex evolutionary history of the origin of the guanidinium toxins, the functional role and putative interactions of these ion channel blockers in cyanobacteria versus dinoflagellates remains an intriguing question. In contrast to the PST-producing cyanobacteria, which are typically found in fresh or brackish waters, the toxigenic dinoflagellates are exclusively marine, and there is little evidence for an important role for these toxins in Na^+^ ion equilibration in response to salinity or pH shifts. In fact, the multiple hypotheses for the putative chemical ecological role of PSTs in dinoflagellates (reviewed by [112]) include sequestration of nitrogen, cell communication, pheromone activity and ion channel regulation, but allelochemical defense against grazers or microeukaryote competitors is most often invoked. If chemical defense, particularly against metazoan grazers, is indeed the primary function of these compounds in dinoflagellates, this implies that the evolutionary function and selective pressure on the biosynthetic genes may already have diverged from the cyanobacteria. In that case, the potent effects on the Na_V_ ion channels of the highly evolved neuromuscular systems of marine mammals, sea birds, teleost fish and human consumers of toxic seafood must be viewed as “collateral damage” in the chemical arms race—but not of evolutionary adaptive significance.

## 7. Guanidinium Toxins as Ion Channel Research Subjects and Tools

### 7.1. Diagnostic Detection Assays for Guanidinium Toxins

Purified guanidinium toxins were first used as research tools to investigate the properties of ion-conducting membrane proteins, but are now more widely employed for calibration of chemical instrumental analysis and assay detection methods for food safety surveillance programs. Chemical analytical methods, based on the physicochemical characteristics of the molecules, are rapidly replacing the intraperitoneal mouse bioassay (MBA) [113,114] for routine screening of guanidinium toxins [115]. Such instrumental analytical methods are, in general, time consuming, and require expensive equipment and infrastructure, and a high level of technical expertise, but they provide specific qualitative and quantitative information on the toxin composition and chemical structures. The major drawbacks are that novel toxins and unknown toxin analogs will be missed by these techniques, and the lack of a complete spectrum of all potential toxins for instrumental calibration (and hence knowledge of their specific potency) hampers accurate calculated estimates of toxicity.

Biological and biochemical in vitro assay methods for guanidinium toxins offer an alternative to whole animal bioassays and chemical analytical techniques albeit with only semi-quantitative detection [116,117]. Diagnostic assays for guanidinium toxins can be sub-divided into two general categories, depending upon their mode of detection: functional assays, such as the receptor binding assay (RBA), reflect the relative affinity of the toxin analogs and mode of action for in vivo systems, whereas structural assays, such as immunoassays and enzyme-inhibition assays, are based upon biochemical recognition of structural features of the toxin analogs that may or may not correlate well with respective ion-channel binding affinities. For routine toxin monitoring programs these assay methods are often configured as rapid diagnostic kits, and used only for screening presence/absence of the toxin group or to indicate when toxin levels exceed the regulatory limit. Such in vitro assay techniques can be configured in a parallel and/or multiplex format for high throughput screening of putatively toxic samples. Typically, application and interpretation of the assay techniques does not require a high level of technical expertise or large capital investment in diagnostic detection systems [118]. While there is intensive research on optimization and configuration of alternative assay formats for routine toxin screening of toxin contaminated seafood, the role of diagnostic assays for elucidation of toxin binding mechanisms and ion channel research is rather more limited. One disadvantage of these methods is that it is not possible to differentiate among toxins that share the same mechanism of action, and thus the identity of the active molecules will remain unknown. Detection of different guanidinium analogs may relate to their specific ability to interact with the receptor, but in most cases, these tools will only provide an overall estimate of general toxicity.

The receptor binding assay (RBA) for guanidinium toxins was originally developed as a specific research tool for determination of toxin kinetics and estimation of Na_V_ channel densities in excitable membranes. Given the need for simple and sensitive methods for detection of guanidinium toxins, attention was directed to development of alternative RBA techniques based upon NaV ion channel activation/blockage by applying pharmaceuticals. Veratridine—a neurotoxic alkaloid from plants of the Liliaceae family—was known to cause persistent activation of Na_V_ channels, but this activity was shown to be blocked by TTX [119,120]. Application of either TTX or STX counteracted the effect of ouabain inhibition of Na^+^-K^+^ ATPase in neuroblastoma cell membranes by blocking the Na_V_ channel [121]. With this knowledge, Kogure et al. [122] developed an assay for guanidinium toxins which involved veratridine to activate Na_V_ channels and ouabain to inhibit the effect on Na^+^-K^+^ ATPase in neuroblastoma cells. This treatment resulted in visible cell swelling and rounding up, with loss of osmoregulatory function leading to eventual cell lysis. This uncontrollable increase in intracellular Na^+^ concentration was counteracted by the inhibitory effect of TTX or STX in a concentration-dependent manner.

Later refinements of the Neuro-2A neuroblastoma cell toxicity assay into a microwell plate format for scanning absorbance of a stain (crystal violet) incorporated into intact neuroblastoma cells showed high comparability to the AOAC mouse bioassay, but with higher sensitivity, for PST-containing extracts of dinoflagellates (*Alexandrium* spp.) and shellfish tissues [123]. A similar format demonstrated suitable applicability for semi-quantitative rapid screening and detection of PST analogs produced by cyanobacteria, with good correlation with chromatographic methods for known toxins [124]. An alternative fluorimetric microplate assay as a quantitative RBA employs a fluorescent stain (bis-oxonol), with toxin-dose dependent inhibition of depolarization by veratridine [125].

In recent configurations, the RBA has been designed as a competitive radioligand binding assay with a radio-labelled active molecule (e.g., STX or TTX) applied to a rat brain membrane preparation containing a known number of available receptor sites. With this approach, competitive detection has been achieved with ^3^H-labelled guanidinium toxins (usually STX or TTX) [117,126,127]. The extremely high affinity of guanidinium toxins for their molecular target (in the nanomolar range) is usually directly proportional to their in vivo toxic potency, yielding good estimates of their respective toxicity. An evaluation of binding affinities of different STX analogs yielded toxin potency estimates that closely tracked those calculated with the AOAC mouse bioassay [128] and this functional assay is now an AOAC certified detection method for STX [129]. The use of 11-[^3^H]-TTX has also been validated as an alternative radio-ligand to STX in a heterologous receptor binding assay for PSTs [130]. Modifications to the original technique have increased the efficiency of the assays by automated scintillation counting in individual vials [131] or with microplates [132].

The radio-label approach remains the most common RBA configuration for guanidinium toxins, but the availability of ^3^H-labelled ligand has not always been consistent. The requirement for radioisotopes and handling facilities for their use has been an impediment for many laboratories and jurisdictions, although the International Atomic Energy Agency (IAEA) continues to provide global support and encouragement for the application of this method, particularly in developing countries where analytical instrumentation and technical expertise for toxin analysis is often lacking.

### 7.2. Biomedical and Therapeutic Application of Guanidinium Toxins

Natural toxins and venoms have been used in traditional remedies since ancient times, but research on the specific potency, mode of action and secondary effects of natural bioactive molecules as pharmaceuticals has developed only within the last 60 years. The intensive research on guanidinium toxins as potential therapeutants has focused on their ion-channel blocking activity, but the scope has been limited due to their known high acute toxicity [45] and relative lack of knowledge on chronic effects.

Chronic pain treatment has been declared a global health priority, but the multivariate causes, such as cancer, diverse nerve injuries or nerve damage, rheumatoid arthritis, among others, creates a complexity of syndromes that is difficult to treat. Conventional pain control medications all have potentially critical secondary effects, especially when administered in high dosages and for extended periods. In particular, natural and synthetic opioids have been increasingly prescribed to manage long-term chronic pain, but this has led to higher rates of opioid use disorders and drug abuse [133,134], as well as overdose deaths [135,136]. Nevertheless, for some patients, opioids are the only viable pain-management medications at this time.

The functional effects of guanidinium toxins on ion-channel blockage and desensitization have guided potential application in pain management. Given the fact that they do not cause myocardial depression or arrhythmias because they do not bind significantly to Na_V_ 1.5 channels, the main sodium channel in the heart [137], allows the possibility of effective application in pain management. Furthermore, these toxins have been shown to be benign to nerves and muscle tissues [138], and there is no evidence of acclimation that could lead of addiction as with opioids, providing additional encouragement for use in pain control strategies.

Some studies in rabbits have demonstrated that the application of TTX produces local anesthesia that allowed corneal surgery in rabbits [139], and for systemic analgesic treatments for long-term pain with neuropathic features [137,138,140]. In combination with the local anesthetic bupivacaine or the neurotransmitter epinephrine, these drugs reduce the systemic toxicity of TTX and hence the lethal dose but also yield an intensification of its potency for sensory blockade [137]. Nevertheless, in spite of evidence of only mild toxicity after administration for pain relief for longer than two weeks, results were positive for only 17 of 31 human patients [141]. This indicates that the mechanisms of the analgesic effect and the causes of individual variation remain poorly understood.

Certain purified STX analogs have also been applied as therapeutants and have shown considerable success in recent medical trials. An epimeric mixture of gonyautoxins 2 and 3 (GTX 2/3) (see Figure 2 for reference) has been used in the treatment of chronic and acute anal fissures, by producing a flaccid paralysis of the anal muscle in a safe and effective way, and thereby reducing healing time [142,143]. This same GTX mixture was applied to treat tension-type headache (TTH) and as an anesthetic for total knee arthroplasty (TKA). For TTH, the treatment was by intramuscular injections, causing an immediate muscle relaxation and pain reduction that lasted for more than 2 months in some cases [144]. Pain blockage for TKA was achieved by local infiltration, and there were no side effects or adverse reactions without the use of opioids [145]. Neosaxitoxin, an N-1 hydroxylated STX analog (see Figure 2), has been used in the treatment of achalasia, a gastrointestinal motility disorder resulting from a failure of the lower esophageal sphincter that causes dysphagia or chest pain. Local application of this toxin in small amounts caused sphincter relaxation and paralysis of the muscle, lasting for at least two days [146]. The same analog has proven effective as a local anesthetic and as a long-acting pain blocker in the treatment of bladder pain syndrome, by local infiltration of small doses into the bladder submucosa with no adverse secondary effects over 90 days [147,148,149].

Apart from pain control, guanidinium toxins have also been explored as potential treatments of unregulated drug dependence. Studies with rats have found that microinjections of TTX into specific brain regions decreased the demand for cocaine-induced stimuli, and helped to increase knowledge regarding activity and function of drug dependence mechanisms and related brain structures [150]. Recent evidence from experiments on morphine-dependent mice and rats has suggested that TTX could be valuable in the treatment heroin-dependent human patients [151]. In this fashion, guanidinium toxins could serve not only as alternatives to opioids in pain management, but also to reduce craving and dependence of chronic opiate users.

### 7.3. Molecular Bioinformatics

**Figure 7:**
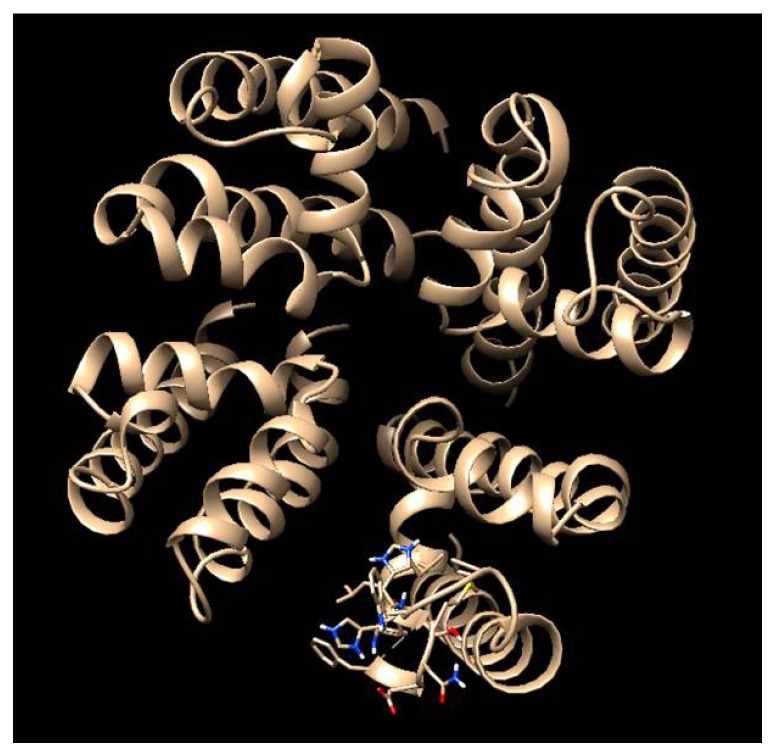
External vestibule of an Na_V_1.4 homology model with P-loops from domains I–IV [39]. Image created with VMD 1.9.1 software [152].

Computational approaches and imaging tools are being developed and implemented as valuable techniques for research on ion channels to decipher their ultrastructure, and to understand the binding properties of guanidinium toxins to their molecular target [39]. To some extent, the development of in silico bioinformatic tools reflects the necessity to restrict the use of live animal models as test subjects because of heightened international concerns for animal rights, and also due to high maintenance and certification costs for facilities where the experimental animals are kept. Beyond these logistical constraints, there are also clear technological advantages in applying novel molecular imaging tools, docking and molecular dynamics (MD) as virtual diagnostic probes and to generate testable hypotheses regarding structural-functional relationships to toxin potency. Access to images generated with great accuracy through the use of specialized computational software has led to a better understanding of the three dimensional structures (Figure 7). In the near future these relatively new analytical tools will even provide specific information at molecular and atomic levels, such as molecular interactions and mechanisms involved in toxicity.

The accuracy of models for computational studies is dependent upon their characteristics to yield precise free energy calculations [153]. Several Na_V_ models have been proposed to clarify the mode of action of guanidinium toxins and to interpret experimental data with molecular accuracy [154,155]. These approaches have shown a wider pore on Na_V_ channels when compared to K^+^ channels, and this could partially explain why Na_V_ channels provide access of guanidinium toxins to the selectivity-filter residues [154]. Bioinformatic approaches have already been applied to explore hindrance effects and electrostatic properties among protein residues and toxins. Yet at present there is still no validated mammalian model for the Na_V_ channel, simulations have been therefore typically performed with homology models based on crystal structures of bacterial Na_V_ channels [153,154], but these are not direct homologs and have a distinct evolutionary origin. Research on crystal structures of mammalian ion channels and construction of accurate models is essential because of the structural discrepancies from bacterial analogs. Unfortunately, there are few crystal structures of such membrane proteins.

In spite of the lack of Na^+^ channel structural models for humans, rapid advancements in molecular studies of homologs from other animal nervous systems, such as insects (e.g., cockroach) and the electric eel have generated novel structural and functional insights. The structure of an Na_V_ from a eukaryotic organism, the American cockroach *Periplaneta americana*, has now been fully elucidated [156], and this will allow the construction of more accurate models for vertebrates. A recent review of Na^+^ ion channel structural aspects in insects as related to insecticide resistance [157] provides a useful basis for comparison with mammalian systems. In the electric eel *Electrophorus electricus* the coding cDNA sequences of voltage-gated Na^+^ channel (*scn*) α-subunit (*scna*) and β-subunit (*scnb*) isoforms have been determined, and the quantification of the mRNA transcript levels in the main electrogenerative organs served to elucidate differential functional aspects among electrocytes from different tissues [158]. Although the objectives of such Na^+^ ion channel studies on insecticide resistance and electrogenesis for predation, defense and sensory detection, respectively, are not strictly applicable to the interpretation of structural-functional interactions of Na^+^ blocking toxins in mammals, they could provide templates for molecular bioinformatic modelling following application of guanidinium toxins, in particular.

Molecular docking tools can reveal the mechanisms involved in binding recognition in great detail, and in a way that cannot be deduced merely from electrophysiological research [159]. This approach may allow fast screening of binding interactions with a given protein for many guanidinium analogs in short time. As Na_V_ bioinformatic models become more accurate, the information obtained with this approach will be more precise as well. Precision is currently limited [160] because of simplifying assumptions and the lack of inclusion of several biological parameters, such as lipid membrane interactions or water barriers. The protein is unrealistically considered as a static structure, with only the toxin as a dynamic molecule. Optimal free energy (∆G) values are calculated from scoring function algorithms that depend upon the bonding or non-bonding interactions between the ligand and the target [161]. With respect to Na^+^ channel blocking activity, this approach has been applied mainly to μ-conotoxins [162], small disulfide-rich proteins comprising 14–20 amino acid residues, but which are structurally and biosynthetically unrelated to the guanidinium toxins. Tikhonov and Zhorov [155] have performed docking simulations on Na_V_ channel 1.4 with TTX and µ-conotoxins to predict their optimal orientation and the interaction energy consistent with experimental results; their analysis suggested that Na^+^ channels are more similar to K^+^ channels than expected. This approach has also provided theoretical information about ligand properties and molecular interactions between STX and many of their naturally occurring analogs, and the same isoform of the Na_V_ channel [39].

Molecular dynamics (MD) is an alternative approach that evaluates different structural conformations to provide more precise information, although this approach increases computational costs and is more time consuming than molecular docking simulations. The first pioneering MD simulation with the peptide neurotoxin I (SH1) from the sea anemone *Stichodactyla helianthus* was conducted in 1991 [163]. A later important step in MD application to Na_V_ channel research was the generation of homology model simulations of the Na_V_ channel recognition properties towards the tarantula peptide toxin psalmotoxin-1 [164], which can bind to a particular isoform (ASIC1) of the Acid Sensing Ion Channel. Although ASICs are proton-gated Na^+^ channels and open when H^+^ binds, unlike the Na_V_ channels involved in recognition of guanidinium toxins, the general MD modelling approach should be applicable for these non-peptidic toxins. Recent MD simulations have been useful to better understand the human Na_V_1.5 channel. For example, Ahmed et al. [165] developed a homology model refined through MD simulations in the lipid membrane bilayer; they noted the existence of two potential binding sites for the Na^+^ ion at the channel selectivity filter. By employing a more refined technique, called steered molecular dynamics (SMD), they were also able to identify the key residues related to this process, none of these molecular bioinformatic approaches are sufficient to describe the exquisiteness of ion channels and their interactions with ligands (pharmaceuticals, toxins, etc.), but a combination of docking modelling and MD, supplemented with electrophysiological and genetic information could be an effective strategy to explain the binding variability properties and chemical diversity of Na^+^ channel blockers. This would contribute to a better understanding of the structure of the channel and assist in defining the molecular mechanisms for drug interactions in finer detail. Such a comprehensive strategy has yet to be applied to interaction of guanidinium toxins with Na_V_ ion channels.

## 8. Conclusions and Future Perspectives

The basic neurophysiology and mode of interactions of guanidinium toxins with Na_V_ ion channels have been explored for many decades and are rather well understood. Nevertheless, there remain incremental challenges to scientific knowledge and progress, from the ongoing search for new natural sources and biosynthetic mechanisms for known and novel structures to potential technological applications as research tools, e.g., in diagnostic detection systems, and as pharmacological treatments. Further development and application of advanced chromatographic techniques for toxin analyte separation and enhanced high resolution detection systems, such as mass spectrometry, supported by nuclear magnetic resonance (NMR), will yield structural elucidation of novel guanidinium toxins from cryptic natural sources. Configuration of these analytical systems for surveillance of guanidinium toxins in food safety regimes is, however, dependent on a reliable supply of putative toxins for calibration standards and for specific toxicity assays. The toxicity of many recently discovered guanidinium analogs remains unknown, mostly due to a lack of purified material for analytical standards and toxicity trials.

Research on marine toxins also faces new challenges, such as the expanding ban on the use of animals for toxicity testing in many global jurisdictions. Requirements for research and improved regulatory regimes will lead to further development of analytical standards and instruments, as well as new assay methods, in particular biologically based functional assays, and in silico bioinformatic tools.

Pharmacological applications of guanidinium toxins have a promising future, as already shown by encouraging results for pain treatment and wound healing. The exquisite specificity of these toxins for certain Na_V_ channels, and the relative lack of adverse acute or chronic secondary effects, makes them valuable molecules for analgesic and anesthetic purposes.

The biggest challenge is to elucidate and understand the cellular functions of guanidinium toxins within the producing organisms, and to determine their eco-evolutionary significance. The evolution of Na_V_ channels preceded the development of neurons, and presumably were permeable to both Na^+^ and Ca^2+^ in early metazoans [93]. Yet the co-evolution (if any) of voltage gated ion channels and biosynthetic genes for guanidinium toxins in bacteria, cyanobacteria and marine dinoflagellates remains a mystery. To unravel the details, functional assays of Na_V_ channel homologs would help to elucidate biological functions and the evolutionary pathway of Na_V_ channels. How Na^+^ selectivity could have evolved from Ca^2+^ (and perhaps even earlier from K^+^) selectivity by sequential mutations will require further explorations on ion selectivity of these respective channels [92]. Better knowledge of the Na_V_ channel function will open the door to generation of more accurate hypotheses on ligand binding interactions of toxins with these respective proteins. After bioinformatic refinements in the near future, accurate prediction of specific toxicity of known and novel guanidinium analogs will be feasible with such in silico analyses.

Given that predicted climatic changes may favor proliferation and range extension of toxigenic marine microalgal and freshwater cyanobacterial blooms through increased water temperatures and other related climate-driven properties of aquatic systems, it is of critical importance to achieve an ecological understanding of toxigenic mechanisms. Anthropogenic factors [31,165], including increased nutrient loading, may enhance chronic exposure to guanidinium toxins via food or drinking water. The issue of sub-lethal toxin exposure deserves special attention, particularly in the context of pollution and nutrient loading of freshwater systems for drinking water for humans and livestock. The serious impacts on human health, aquatic resources and ecosystem functioning caused by guanidinium toxins require deeper insights into the origin and spreading of the biosynthetic genes for STX and TTX analogs in both prokaryotes and eukaryotes. Now that the responsible gene clusters have been identified, and the biosynthetic pathways have been elucidated, at least for the STX group, metagenomic and metatranscriptomic analysis will provide the basis for a more reliable predictive risk assessment.

## Figures and Tables

**Table 1:** Relative acute toxicities of STX (=1.0) and some derivatives according to intraperitoneal mouse bioassay (IP MBA) data compiled by Durán-Riveroll et al. [39] with values calculated from * Sullivan et al. [40] and ** Oshima [41]. Relative oral toxicity values in mice by voluntary feeding/gavage are according to Munday et al. [42].

Toxin Analog	Relative Toxicity IP MBA	Relative Toxicity Voluntary Feeding/Gavage
STX	1.0	1.0
NEO	1.0	2.5/1.7
GTX1	1.0	
GTX4	0.7	
GTX1/4		0.9/0.7
GTX2	0.4	
GTX3	0.6	
GTX2/3		0.6/0.5
dcSTX	1.0	
dcGTX1	0.5 *	
dcGTX4	0.5 *	
dcGTX2	0.2	
dcGTX3	0.4	
dcGTX2/3		0.1/0.2
dcNEO	0.4	0.2/0.2
B1	0.1	0.06/0.05
B2	0.1	<0.02/0.04
C1	0.01 **	
C2	0.1	
C1/2		0.04/0.03
C3	0.01 **	
C4	0.1	

**Table 2:** Acute toxicity of guanidinium toxins and the Na_V_ blocking protein µ-conotoxin (for reference) in different animal models and via various routes of administration. LD_50_ is defined as the dose that causes death of 50% of the test subjects under the specified administration conditions. * intramuscular injections were intended to directly expose the sciatic nerve to STX [43]. O, oral; i.v., intravenous; i.p. intraperitoneal; i.m., intramuscular; s.c., subcutaneous; i.g., intragastric; O/G, oral/gavage; O/F, oral/feeding.

Animal Model	Toxin	Route of Administration	LD_50_ (nmol kg^−1^)	Reference
Pigeon	STX	O	302	[43]
Rabbit		i.v.	1	
		O	601	
Rat		i.p.	35	
		i.m. *	23	
		O	638	
Cat		O	844	
Chicken		i.v.	1	
Dog		O	601	
Guinea pig		O	449	
Mouse		i.v.	11-28	
		s.c.	43	
		i.p.	26-33	
	NEO	i.p.	8.9	[42]
	dcSTX	i.p.	35.4	
	GTX1/4	i.p.	14.6	
	GTX1/3	i.p.	36.7	
	STX	O/G	1190	
		O/F	3200	
	NEO	O/G	700	
		O/F	1260	
	dcSTX	O/G	2600	
		O/F	8680	
	GTX1/4	O/G	1610	
		O/F	3420	
	GTX2/3	O/G	2230	
		O/F	5590	
	TTX	i.v.	28	[44]
		i.p.	34	[45]
		s.c.	39-50	[45,46]
		i.g.	1668	[45]
		O	727	[47] [47]
	11-deoxyTTX	i.p.	231	
	µ-conotoxin GIIIA	i.p.	1066	[48]
	µ-conotoxin GIIIB	i.p.	345	

**Table 3:** Classification of Na_V_ channel isoforms according to their sensitivity to TTX [72,73,75]. CNS, central nervous system; PNS, peripheral nervous system; SMCs, smooth muscle cells; DRG, dorsal root ganglion. IC_50_ (half maximal inhibitory concentration) is the dose that inhibits by 50% the biological function under the specified administration conditions.

Na_V_ Channel Isoform	Predominant Location	TTX IC_50_ [nM]	Sensitive/Resistant
Na_V_1.1	CNS, PNS, Heart	5.9	Sensitive
Na_V_1.2	CNS	7.8	Sensitive
Na_V_1.3	CNS (embryonic)	2.0	Sensitive
Na_V_1.4	Skeletal muscle	4.5	Sensitive
Na_V_1.5	Heart, CNS	1970	Resistant
Na_V_1.6	CNS, PNS, SMCs, DRG	3.8	Sensitive
Na_V_1.7	PNS, DRG	5.5	Sensitive
Na_V_1.8	PNS, DRG	1330	Resistant
Na_V_1.9	PNS, DRG	59,600	Resistant
